# Detection of Yellow Fever Virus in Sylvatic Mosquitoes during Disease Outbreaks of 2017–2018 in Minas Gerais State, Brazil

**DOI:** 10.3390/insects10050136

**Published:** 2019-05-10

**Authors:** Guilherme Garcia Pinheiro, Marcele Neves Rocha, Maria Angélica de Oliveira, Luciano Andrade Moreira, José Dilermando Andrade Filho

**Affiliations:** 1 Coleção de Mosquitos Neotropicais, Instituto René Rachou, Avenida Augusto de Lima, 1715, Belo Horizonte 30190-002, Brazil; angelica.oliveira@fiocruz.br; 2 Mosquitos Vetores: Endossimbiontes e Interação Patógeno-Vetor, Instituto René Rachou, Avenida Augusto de Lima, 1715, Belo Horizonte 30190-002, Brazil; marcele.rocha@fiocruz.br (M.N.R.); luciano.andrade@fiocruz.br (L.A.M.); 3 Grupo de Estudos em Leishmanioses, Instituto René Rachou, Avenida Augusto de Lima, 1715, Belo Horizonte 30190-002, Brazil

**Keywords:** arboviruses, yellow fever, mosquitoes

## Abstract

Brazil has experienced several arbovirus outbreaks in recent years, among which yellow fever stands out. The state of Minas Gerais faced outbreaks of sylvatic yellow fever in 2017 and 2018, with 1002 confirmed cases and 340 deaths. This work presents the results of survey efforts to detect the yellow fever virus in mosquitoes from two conservation areas in the metropolitan region of Belo Horizonte, Brazil. A total of 867 mosquitoes of 20 species were collected between September 2017 and May 2018, the most abundant being *Psorophora* (*Janthinosoma*) *ferox* (von Humboldt, 1819) (31.3%), *Limatus durhamii* Theobald, 1901 (19.1%) and *Haemagogus* (*Haemagogus*) *janthinomys* Dyar, 1921 (18.2%). Total RNA was extracted from the mosquitoes for real-time PCR analysis for yellow fever, chikungunya, mayaro, Zika and dengue viruses. The yellow fever infection rate was 8.2% for *Hg. janthinomys* (13 mosquitoes), which is the main vector of sylvatic yellow fever in Brazil. In addition to surveying the mosquito fauna of these conservation units, this work demonstrates the importance of monitoring the circulation of viruses near large urban centers.

## 1. Introduction

Yellow fever (YF) is a severe acute disease with high lethality (20–50% among symptomatic cases) [1] and is caused by yellow fever virus (YFV), an arbovirus of the family *Flaviviridae*. The virus is exclusively transmitted to its hosts through bites of female mosquitos of some species of the family Culicidae [2,3]. The disease presents two distinct transmission cycles: the sylvatic cycle and the urban cycle. Although these cycles share similarities regarding their etiology and disease evolution, they have different vectors and hosts. The main vector for the sylvatic cycle in the Americas is *Haemagogus* (*Haemagogus*) *janthinomys* Dyar, 1921, which is widely distributed in Brazil from the state of Santa Catarina in the south to the North Region of the country [4]. The main hosts of the virus are non-human primates (NHP’s), such as howler monkeys (*Alouatta* sp), while humans become infected accidentally. The main vector of the urban cycle is *Aedes* (*Stegomyia*) *aegypti* (Linnaeus, 1972), however there have been no reports of urban yellow fever in Brazil since 1942 [5,6]. On the African continent, however, the urban cycle is responsible for most of the human cases of YF recorded annually [2].

According to WHO [7], portions of 47 countries are considered endemic areas for YF, 34 of which are in Africa and 13 in Latin America, with 90% of the reported cases being concentrated in Africa. Between the end of 2016 and the middle of 2018, two of the largest outbreaks, in terms of cases, in the last 50 years were recorded in Brazil, with the Southeast Region being the most affected [6,8]. A total of 779 cases were confirmed with 262 deaths in 2016/2017, and 1376 confirmed cases with 483 deaths in 2017/2018 [8]. This increase in the number of cases may be related to factors such as low vaccination coverage and viral circulation in regions where it previously did not occur as well as changes in the viral genotype [9,10].

In addition to YF, Brazil has experienced outbreaks of other arboviruses during 2017–2018, including Zika, for which there were 3308 confirmed cases, chikungunya with 61,625 confirmed cases and dengue with 145,137 confirmed cases and 304 deaths [11]. Another arthropod-borne disease that has come to attention is mayaro virus disease, for which the majority of cases have been registered in the Northern Region of the country where it is endemic, although it has also been detected in other regions of Brazil [12].

The objective of this study was to survey the mosquito fauna of two conservation areas in the metropolitan region of Belo Horizonte, the capital of the state of Minas Gerais, Brazil, and to evaluate the viral presence of yellow fever virus (YFV), dengue virus (DENV), chikungunya virus (CHIKV), Zika virus (ZIKV) and mayaro virus (MAYV).

## 2. Materials and Methods 

### 2.1. Study Area

The study was performed in Parque Estadual da Serra do Rola-Moça (PESRM; Serra do Rola-Moça State Park) and the Estação Ecológica de Fechos (EEF; Fechos Ecological Station), which are located in four municipalities (Belo Horizonte, Brumadinho, Ibirité and Nova Lima) in the metropolitan area of Belo Horizonte. The two reserves are located in an area of transition between the Brazilian Savannah (Cerrado) and Atlantic Forest biomes and possess areas with human occupation at their borders. The forest areas of the region are classified as seasonal semideciduous forest with trees of medium and high height. The climate is typically tropical with two distinct seasons; a rainy season between September and March, and a dry season during the rest of the year with little precipitation and low humidity [13].

**Figure 1:**
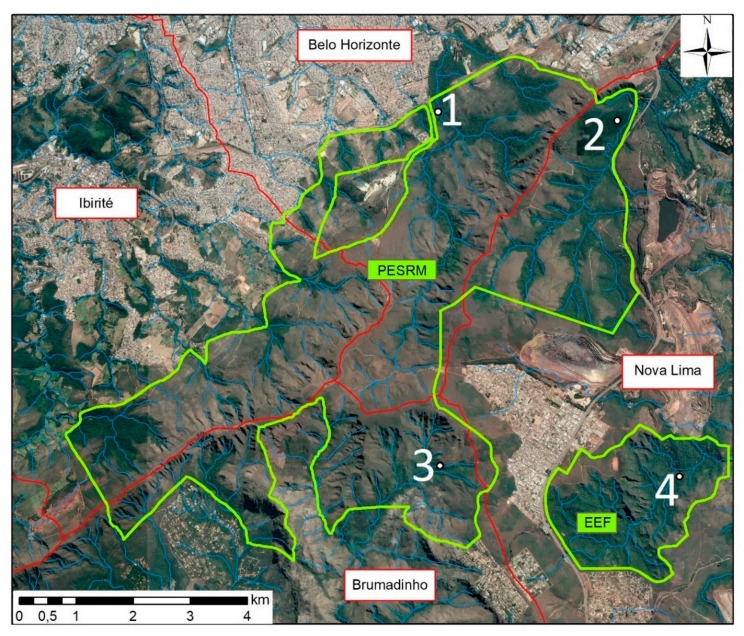
Mosquito sampling areas (1 to 4) of Parque Estadual da Serra do Rola-Moça (PERSM) and Estação Ecológica de Fechos (EEF) and the nearby urban areas. Adapted from Instituto Estadual de Florestas [13]. The red lines represent the territorial limits between the municipalities, the green lines delimit the study area, the blue lines represent the water courses present in the region and the white dots represent the collection areas.

Three sampling areas were selected in PESRM and one in EEF (Figure 1). All four sampling areas were located in dense forest with little wind incidence, close to watercourses and 150 m to 1.5 km from inhabited areas. Area 1 (44°00′12″ W, 20°00′12″ S) was located 150 m from the district of Barreiro in the city of Belo Horizonte; Area 2 (43°58′12″ W, 20°00′47″ S) was located 1 km from a residential condominium in the municipality of Nova Lima; and Area 3 (44°00′02″ W, 20°04′05″ S) was located 1.5 km from a district of the municipality of Nova Lima and 3 km from a district of the municipality of Brumadinho. Area 4 (43°57′53″ W, 20°04′21″ S) was the only sampling area located in EEF and was selected because it is located near residential condominiums of the municipality of Nova Lima, however it differs from Area 2 as it has a larger area of forest. The location of each point was obtained through the use of Global Positioning System (GPS) (GPSMAP^®^ 62s, GARMIN, Olathe, KS, USA).

### 2.2. Mosquito Collection and Identification

Mosquitoes were collected in September and December of 2017 and in February, March and May of 2018. Due to low yield and operational problems, the February collection was repeated in March. Daytime collections were carried out in the afternoon with a manual aspirator, an entomological net and automatic traps (HP model) [14] with CO_2_ lures (dry ice). Night collections were carried out only with automatic traps baited with light and CO_2_, which were withdrawn the following morning. Two automatic traps were placed approximately 1.5 m off the ground with 20 m distance between them in all four points. All mosquitos were collected at ground level. Climatic conditions were recorded for all collections using thermohygrometer (Icoterm, Porto Alegre, Brazil). Collected mosquitoes were placed in individual tubes and stored in a styrofoam box with dry ice (to preserve viral RNA) before storage in a −80 °C freezer.

The objective of the first field collection (September) was to perform a detailed survey of the mosquito fauna. Thus, all collected mosquitoes were mounted on entomological pins and identified to species or genus according to the classification proposed by Harbach & Kitching [15] and specific identification keys of the Coleção de Mosquitos Neotropicais (CMN; Neotropical Mosquito Collection) of the Instituto René Rachou (IRR; René Rachou Institute). These specimens served as a reference to accelerate the identification of mosquitoes collected in subsequent field trips in order to conserve any viral RNA present in mosquitoes. Species that were not sampled during the first field trip were thawed and mounted. All mounted mosquitoes were ultimately deposited in CMN. Brazilian government license: SISBIO nº 57712-1 and SISGEN n° A72F743.

### 2.3. Virus Detection

Mosquitoes were individually macerated in screwcap tubes (Sarstedt, Nümbrecht, Germany) containing a glass bead (Sigma, Tokyo, Japan) and 150 μL of PBS for 1 min and 30 s using a minibeadbeater machine (Biospec Products, Bartlesville, OK, USA). RNA extraction was performed with 50 μL of macerated mosquito using 50 μL of squash buffer solution [Tris 99% (Inlab, São Paulo Brazil), sodium chloride (Synth, Diadema, Brazil), EDTA (Synth, Diadema, Brazil)] and added Proteinase K (Qiagen, Hilden, Germany) 0.6 μL in Hard-Shell^®^ 96-Well PCR Plates (low profile, thin walled, skirted, and white/clear) (BioRad, Hercules, CA, USA). Extraction plates were heated on a thermocycler (Applied Biosystems, Foster City, CA, USA) at 56 °C for 5 min, followed by 98 °C for 15 min, and then were stored at 12 °C until PCR. In this type of extraction, there is no need for sample purification; in the final solution, there is RNA and DNA from the extracted material [16].

Detection of viral RNA was performed via Real-time PCR (RT-qPCR) using the Gotaq^®^ Probe 1-step RT-qPCR System (Promega, Madison, WI, USA) according to the manufacturer’s protocol. Multiplex reactions were performed with the primers and probes described in Table 1.

Multiplex reactions targeted YFV, CHIKV and DENV in the first reaction, and ZIKV and MAYV in the second reaction, and were performed using a Lightcycler96 real-time PCR machine (Roche, Basel, Switzerland) with the following standard cycling conditions: reverse transcription at 45 °C for 15 min, reverse transcriptase inactivation and Gotaq^®^ DNA Polymerase (Promega, Madison, WI, USA) activation at 95 °C for 2 min and initial denaturation annealing and extension for 40 cycles at 95 °C for 15 s followed by 60 °C for 1 min. The reaction volume was 10 μL: 2× RT-PCR Reaction Mix (Promega, Madison, WI, USA), RT-enzyme solution (Promega, Madison, WI, USA), 1 μM YFV primer (For + Rev) (IDT, Coralville, IA, USA), 0.2 μM YFV probe (IDT, Coralville, IA, USA), 0.5 μM other primers (IDT, Coralville, IA, USA) and 0.1 μM other probes (IDT, Coralville, IA, USA). A fraction (1/40) of the total isolated RNA was used in the reactions. 

RNA/DNA free water was used as the negative control, whereas for the positive control, a YFV gene fragment (5’-NC [17]) cloned into the pGemP plasmid (Promega, Madison, WI, USA) was used. The quantification of viral RNA from YFV infected mosquitoes were performed in comparison with serial dilution (from 10^7^ to 1 copy of viral RNA) of a standard curve (Table S1) of the same gene used as the positive control. Therefore, it was possible to calculate the number of RNA copies per sample [18]. The limit of detection was on the order of 100 copies that corresponded to a Ct value of 36.

### 2.4. Virus Isolation

All YFV positive samples (n = 13) were selected for viral isolation. The non-extracted part of the material that was stored at −80 °C was centrifuged for 2 min at 1500g at 12 °C. Then, 20 μL of the supernatant was inoculated into 25 cm^2^ T-bottles (Sarstedt, Nümbrecht, Germany) prepared with an *Aedes albopictus* C6/36 cell culture (2 × 10^6^ cells per bottle) to isolate the virus. Adsorption was carried out for 1 hour at 28 °C and then 5 mL of L15 medium (Gibco, Thermo Fisher Scientific, Waltham, MA, USA), supplemented with 5% fetal bovine serum (Gibco, Thermo Fisher Scientific, Waltham, MA, USA) and Penicillin Streptomycin (Pen Strep) (Gibco Thermo Fisher Scientific, Waltham, MA, USA) at 10 μg/mL was added [23]. The bottles were maintained for up to 10 days at 28 °C. As the negative control, the same protocol was performed with C6/36 cells only with L15 medium (Mock).

Samples were collected from the bottles individually after seven and 10 days of inoculation and RNA was extracted using the same methodology described above for the squash buffer solution. Next, RT-qPCR for YFV was performed, using the primers described in Table 1, in a singleplex reaction to confirm viral isolation for the 13 samples. 

### 2.5. Data Analysis

All statistical analyses were performed using Prism (Graphpad Version 7.04, GraphPad Software, San Diego, CA, USA). The rate of infection per species in each collection area was calculated by dividing the number of positive mosquitoes by the total number of mosquitoes of the same species collected in the same area. Total infection rate per species was calculated by dividing the number of infected specimens of a species by the total number of specimens of that species.

## 3. Results

A total of 867 mosquitoes were collected, of which 89 were mounted to facilitate subsequent species identification and 778 were frozen and processed for viral RNA detection. As a result, 40 specimens were identified to the level of subgenus, six to the level of genus and the remainder to the level of species. A total of 20 mosquito species of 10 genera were captured (Table 2).

The most abundant species was *Psorophora* (*Janthinosoma*) *ferox* (von Humboldt, 1819) (n = 271; 31.3%), followed by *Limatus durhamii* Theobald, 1901 (n = 166; 19.1%) and *Haemagogus* (*Haemagogus*) *janthinomys* Dyar, 1921 (n = 158, 18.2%).

**Figure 2:**
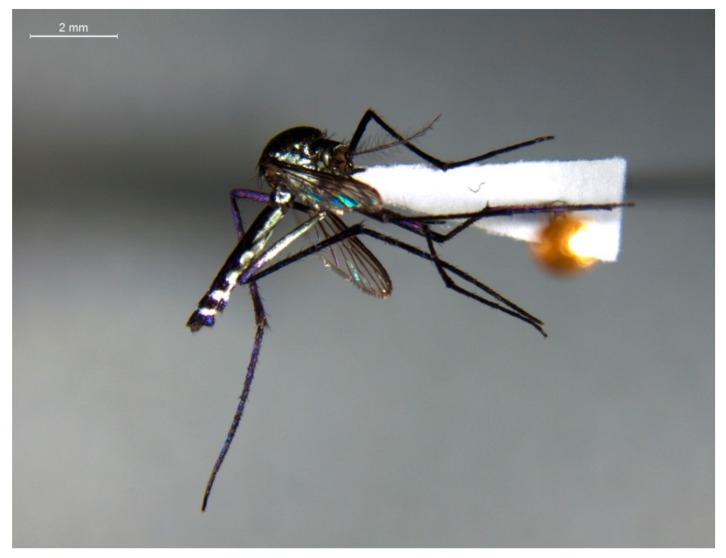
*Haemagogus (Haemagogus) janthinomys* Dyar, 1921, collected in Area 4 in December 2017.

Molecular analyses of the 778 mosquitoes revealed 13 YFV positives, all of which were *Haemagogus janthinomys* (Figure 2). Of the positives, 11 were collected on the same day (January 29, 2018) and in the same sampling area (Area 4). The other two positive mosquitoes were collected in December 2017 in two different areas (Areas 2 and 3). The infection rates for the sampled areas are shown in Table 3 and the Ct value of the samples are available in Table S2 in the supplementary material.

**Figure 3:**
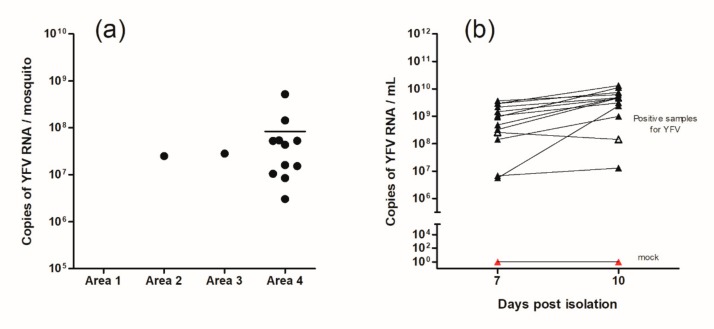
(**a**) Quantification by RT-qPCR of YFV RNA copies per mosquito (*Hg. janthinomys*) by area. Area 1 (PESRM—Belo Horizonte city), Area 2 (PESRM—municipality of Nova Lima), Area 3 (PESRM—Brumadinho municipality) and Area 4 (EEF—Nova Lima municipality). The bar in area 4 represents the mean obtained from the 11 positive specimens (8.42 × 10^7^). (**b**) Quantification of viral copies after seven and 10 days post viral isolation in C6/36 cells. From this, 13 positive samples from the primary screening were isolated. As the negative control, the mock (red triangle) was used. Twelve samples increased the viral amount (full black triangle). A single sample decreased the amount of viral RNA from seven to 10 days (empty black triangle).

Absolute quantification of virus presence in mosquitoes was performed through a standard curve (Figure 3a). Positive mosquitoes possessed, on average, 7.54 × 10^7^ copies of YFV viral RNA. None of the collected mosquitoes were found to possess viral RNA of the other viruses tested (dengue, chikungunya, mayaro and Zika virus).

All YFV RNA-positive samples of the primary screening were isolated and confirmed via RT-qPCR. The number of viral RNA copies per milliliter obtained seven and 10 days after isolation are provided in Figure 3b. Quantification ranged from 10^6^ to 10^8^ copies of viral RNA/mL. Of the 13 isolated samples, 12 presented an increase in virus replication profile after 10 days compared to seven days. Only one sample did not present positive growth after seven days. Cells without supernatant inoculation (negative control—Mock) were indeed negative on RT-qPCR. The Ct value of the isolated samples are available in Table S3 in the supplementary material.

## 4. Discussion

The mosquito fauna collected in the two conservation areas was consistent with the study by Neves [24], who sampled Mangabeiras Municipal Park in Belo Horizonte, which has the same types of soil, climate and vegetation. These three areas are near one another and are connected by ecological corridors. From the 20 species captured in this study, only one (*Hg. janthinomys*) was found to be infected with YFV.

The absolute infection rate of *Hg. janthinomys* was 8.2%, which is similar to the 6.54% minimum infection rate (MIR) found by Dégallier [25] for the same species in Campo Grande, state of Mato Grosso do Sul. Dégallier’s and the present study collected mosquitoes during, or shortly after, human YF cases were reported in the studied areas. Confirmation of the YFV infection through the virus isolation technique confirmed that the viruses detected in primary screening by RT-qPCR were infective particles.

Although three species of *Sabethes* spp. were collected (n = 42 specimens), YFV infection was not detected in any of them. Among the collected species, only *Sabethes* (*Sabethes*) *albiprivus* Theobald, 1903 has been found to be naturally infected by YFV in South America [26]. It is likely that more species of *Sabethes* than those already incriminated play a secondary role in YFV maintenance [5,27].

*Haemagogus* (*Conopostegus*) *leucocelaenus* (Dyar & Shannon, 1924) is considered the main vector of YFV in the South Region of Brazil [5], with most recent studies having detected YFV in this species [28,29] which is more easily found in peri-urban areas due to its greater capacity of adapting to modified environments [30]. Although 42 mosquitoes of this species were collected, viral RNA was not detected in any of them. 

In a study carried out in 2008 in southern Brazil, a YFV infection rate of 1.88% was found for *Aedes* (*Ochlerotatus*) *serratus* (Theobald, 1901) [28]. Although 97 mosquitoes of this species were captured during the present study, none of them tested positive for YFV. Two factors could explain the detection of YFV in *Ae. serratus* infected in Cardoso [28]. The first one could be the presence of viral RNA in the still undigested bloodmeal, and the second would be genetic compatibility between the viral isolate and the *Ae. serratus* population present in that region which could be susceptible to the virus. Further studies are necessary to understand the role of *Ae. serratus* as a YF vector.

The lack of detection of either DENV or CHIKV in the collected mosquitoes, despite recurrent epidemics in the metropolitan area of Belo Horizonte [31], indicates that these two viruses do not seem to be circulating in the wild environments or in other possible vectors in the evaluated period (September 2017 to May 2018). This data suggests that the transmission of these viruses most likely occurs through the bite of *Ae. aegypti*, which is the main vector of these two diseases [32] and circulates mostly in urban areas [5]. The fact that no urban area was selected for this study may have contributed to the non-collection of *Ae. aegypti* specimens. 

Although NHP carcasses were found naturally infected by ZIKV in Minas Gerais between 2016 and 2017 [33], no copies of ZIKV viral RNA were detected in any mosquito collected in the present study and, therefore, further studies are needed to identify if there is a Zika sylvatic cycle occurring. However, since it was not the main objective, no NHP survey in the PESRM in the EEF was performed in this study.

In Brazil, other arthropod-borne diseases with symptoms similar to YF and their transmission through the same vectors can generate confusion in the diagnosis of human cases. In this sense, Costa [12] detected the circulation of MAYV in 34 patients in 2015–2016 in the metropolitan region of Cuiabá in the state of Mato Grosso, while in 2013, Serra [34] found mosquitoes infected with MAYV in Cuiabá, suggesting the circulation of this virus outside the Northern Region of Brazil where it is endemic. *Haemagogus janthinomys* is considered the main vector of Mayaro virus disease [5] and despite the high number of *Hg. janthinomys* collected (n = 158), MAYV was not detected in the present study. Further studies to monitor the circulation of this virus to reduce the risk of possible outbreaks in the Southeast Region, where it may already be circulating, are recommended.

The municipalities where infected mosquitoes were found in the present study—Nova Lima and Brumadinho—had, altogether, 50 YF confirmed cases with 14 deaths during the outbreak period (2017–2018). In addition, NHP epizootics were recorded in January 2018 in Brumadinho and in November 2017 and January 2018 in Nova Lima. Although the three areas where YFV were isolated (Areas 2, 3 and 4) are not as close to the urban perimeter as Area 1, six large residential condominiums are connected to them through ecological corridors, which can easily permit passage of mosquitoes and NHPs. Most of these residential condominiums are located within large areas of Atlantic Forest, which is ideal habitat for species of the genus *Haemagogus* [5].

*Haemagogus janthinomys* is a versatile mosquito. After feeding on a host infected with YFV and after the intrinsic period of infection, it is believed that the species can transmit the virus. Although there are no studies confirming viral transmission, it is considered to be the main vector since it is often found naturally infected at high rates [5,25]. In addition to being infective throughout its life, it has the ability to deposit infected eggs (transovarian transmission), which remain viable for months [5,35]. A mosquito that becomes infected in one of the areas can easily get close to human populations through ecological corridors, making these residential condominiums important areas of risk for YFV transmission and, therefore, they should receive special attention from health authorities.

In addition to the YF risks associated with building houses in forested areas, another activity that is considered to pose YF risks is ecotourism, which is very popular in the metropolitan area of Belo Horizonte due to the large number of preserved areas with trails and waterfalls that attract large numbers of tourists. Therefore, although *Ae. aegypti* have not been found to be infected with YFV during the 2016–2018 epidemic, the risk of re-urbanization of YF exists due to the large number of unvaccinated people who frequent forested areas. Vaccination campaigns in large urban centers is necessary to reduce the risk of outbreaks and the re-emergence of urban YF. 

Yellow fever is a disease with high lethality, yet an effective vaccine is available. If a population has a vaccination coverage of 80%, it is expected to interrupt local YFV transmission [7]. In Brazil, the recommended areas for vaccination covered the north, mid-west and part of the southeast regions and in 2017, more than 4469 municipalities were included in the list. It has been observed that the displacement of the virus appears to be occurring rapidly, spreading to the states of São Paulo and Rio de Janeiro in 2016 and progressing in more areas in these states until 2018.

When new cases were reported in these states, the demand for vaccines increased in regions with high population density. With this, there was a need to increase the production of the vaccine in a short time. An alternative way to address this demand was the use of the fractionated vaccine. Recent studies have already indicated that the fractionated dose (587 IU) promoted similar immunity to the full dose (27,476 IU) [36]. However, this new strategy caused distrust in the population who did not believe that the fractionated dose was effective, thus impacting the coverage below the rates necessary to block local transmission at that moment of epidemics. Several yellow fever vaccination campaigns were promoted and an example of this was observed in January 2017. When the first cases were confirmed in Minas Gerais state, vaccination coverage was 57%, well below the 80% needed to stop local transmission. After intense vaccination campaigns, the coverage reached 90% in October 2018 [37] and no human cases of the disease were recorded from July 2018 to January 2019 [38]. The situation of vaccine coverage in other states was no different [39]. Currently, vaccination coverage reached 55% in the state of Bahia, 71% in Rio de Janeiro and 90% in São Paulo. This success in increasing vaccination coverage may be due to the extension of the campaign promoted by the government [39].

Although São Paulo has reached 90% coverage, YF cases are still being confirmed mainly in the region denominated Vale do Ribeira. In 2019, 86 cases were reported with 23 discarded, 32 confirmed and 31 under investigation. In this period, nine deaths (28.1% of lethality) have already been confirmed. Still in the state of São Paulo, epizootics were reported in 281 municipalities. This shows that despite the intense vaccination campaigns, the virus continues to spread to new regions [40].

One of the factors that could explain the increase in cases of YF is the exacerbated degradation of some natural environments. By losing their natural habitats, some species of NHPs have been forced to change and inhabit modified environments and suffer from overpopulation of small forest patches. In addition, the high density of non-immune NHPs in the Southeast coast, such as marmosets, in forests infested by *Haemagogus* favored the propagation of wild epizootic waves of the YFV to this region and, consequently, human infection. However, further research is needed to understand the dynamic of YFV spread through vast geographic extensions. It has been suggested that the YFV had an average dispersion of about 3 km per day in the Southeast since the beginning of 2017 [10,41]. In a previous outbreak in the South region, the trajectory of yellow fever virus circulation was estimated as 600 km in six months [42].

Associated with YFV dispersion, mosquitoes can disperse the virus over long distances, as demonstrated by Causey et al. [43] when they observed that of females of *Haemagogus* (*Haemagogus*) *spegazzinii* Brèthes, 1912 were collected 11.5 km away from a release point in a few days. These mosquitoes appear to have great dispersal capacity beyond the forests where they emerged from their original larval habitat, a pattern also observed in Central Brazil [44]. This great distance traveled by *Haemagogus* females could be due to a climate with low precipitation which would lead to a reduction in the arrangement of tree holes containing water. In this way, the search for oviposition sites could help in a greater displacement, as well as the search for blood feeding sources for egg maturation. Reduction of NHPs and other wild animal populations as a consequence of the degradation of natural habitat and deaths by YF would cause a reduction of blood sources, inducing the dispersal of the mosquito. Associated with dispersion, it is also interesting to note that wind convection currents around dusk could help these mosquitoes to disperse [39].

Immunization of NHPs could also be a preventive auxiliary form, as by prioritizing the vaccination of confined animals in parks and forest reserves contiguous to urban areas, as well as captive animals in research centers and zoos where the NHP’s population is monitored, the immunization in these cases would be viable. Although this conduct could affect the sentinel role that NHPs play, it also helps limit the exposure of urban mosquitoes to the virus, as well as preserve biodiversity and protect endangered species in conservation areas [39].

## 5. Conclusions

For decades, scientists have warned public health authorities that once the virus started circulating near large metropolitan areas, the risk of re-emergence of YF would increase. Recent studies have revealed that cases of sylvatic YF and infected mosquitoes are being reported closer to large urban areas, such Belo Horizonte and São Paulo. Although no cases of urban YF have been reported in Brazil since 1942, the re-emergence is still a health risk, especially considering that *Ae. aegypti*, the main vector of the urban form of the disease, is distributed throughout the country and the control measures of that vector in peri-urban and urban areas are inefficient. 

Although there have been recent advances in understanding the eco-social determinants involved in the rapid spread of the YFV, several gaps in knowledge still exist. Systematic epidemiological surveillance programs of mosquitoes and NHPs are necessary and would be very effective for monitoring the circulation of the virus. In addition, continued vaccination campaigns would help to keep vaccination coverage at a high level. New NHP’s immunization strategies are also crucial in parks, conservation areas and zoos, protecting endangered species and preserving biodiversity. The results of this study demonstrate the importance of monitoring the circulation of viruses near large urban centers and the search for an effective control and management of these arboviruses to minimize outbreaks such as those that occurred in the last two years. 

## Figures and Tables

**Table 1:** Sequence of primers and probes used in the multiplex RT-qPCR reactions (F = forward primer, R = reverse primer and P = probe).

Virus	Sequence	Reference
Yellow fever virus (YFV)	F-5’/GCT AAT TGA GGT GYA TTG GTC TGC/3’	[17]
	R-5’/CTG CTA ATC GCT CAA MGA ACG/3’	
	P-5’/FAM/ATC GAG TTG/ZEN/CTA GGC AAT AAA CAC/3lAbRQSp/3’	
Chikungunya virus (CHIKV)	F-5’/AAG CTY CGC GTC CTT TAC CAA G/3’	[19]
	R-5’/CCA AAT TGT CCY GGT CTT CCT/3’	
	P-5´/5HEX/CCA ATG TCY/ZEN/TCM GCC TGG ACA CCT TT/3IABkFQ/3´	
Dengue virus (DENV)	F-5´/GGA AGC TGT ACC TTG GTG GTA AGG A/3’	[20] with modifications
	R-5’/CGT TCT GTG CCT GGA ATG ATG/3’	
	P-5’/TEX615/AAC AGC ATA TTG ACG CTG GGA GAG ACC AGA/3IAbRQSp/3’	
Zika virus (ZIKV)	F-5’/TTG GTC ATG ATA CTG CTG ATT GC/3’	[21]
	R-5’/CCT TCC ACA AAG TCC CTA TTG C/3’	
	P-5’/FAM/CGG CAT ACA/ZEN/GCA TCA GGT GCA TAG GAG/3IABKFQ/3’	
Mayaro virus (MAYV)	F-5′/GTG GTC GCA CAG TGA ATC TTT C/3′	[22]
	R-5′/CAA ATG TCC ACC AGG CGA AG/3′	
	P-5′/HEX/ATG GTG GTA/ZEN/GGC TAT CCG ACA GGT C/3IABKFQ/3’	

**Table 2:** Mosquitoes collected between September 2017 and May 2018 in Parque Estadual da Serra do Rola-Moça (PERSM) and Estação Ecológica de Fechos (EEF), Minas Gerais, Brazil.

Taxonomic Category	Sep	Dec	Feb/Mar	May	Total
*Psorophora ferox*	0	25	243	3	271 (31.3%)
*Limatus durhamii*	7	36	118	5	166 (19.1%)
*Haemagogus janthinomys*	0	77	81	0	158 (18.2%)
*Aedes serratus*	0	53	43	1	97 (11.2%)
*Haemagogus leucocelaenus*	0	24	18	0	42 (4.8%)
*Culex* (*Anoedioporpa*) sp	30	0	0	0	30 (3.5%)
*Sabethes purpureus*	6	4	12	2	24 (2.8%)
*Aedes albopictus*	0	8	8	0	16 (1.8%)
*Sabethes petrocchiae*	0	2	7	5	14 (1.6%)
*Aedes terrens*	0	10	0	0	10 (1.2%)
*Aedeomyia squamipennis*	2	2	0	1	5 (0.6%)
*Culex sp*	0	4	1	0	5 (0.6%)
*Culex (Culex*) sp	0	1	1	2	4 (0.5%)
*Culex* (*Melanoconion*) sp	1	0	1	2	4 (0.5%)
*Sabethes albiprivus*	2	0	2	0	4 (0.5%)
*Aedes fluviatilis*	0	0	2	0	2 (0.2%)
*Culex* (*Microculex*) sp	0	0	2	0	2 (0.2%)
*Psorophora albigenu*	0	2	0	0	2 (0.2%)
*Runchomyia cerqueirai*	2	0	0	0	2 (0.2%)
*Wyeomyia fuscipes*	2	0	0	0	2 (0.2%)
*Wyeomyia moerbista*	0	0	2	0	2 (0.2%)
*Anopheles eiseni*	1	0	0	0	1 (0.1%)
*Culex declarator*	0	0	1	0	1 (0.1%)
*Runchomyia reversa*	0	1	0	0	1 (0.1%)
*Wyeomyia alani*	0	0	1	0	1 (0.1%)
*Wyeomyia* sp	0	0	1	0	1 (0.1%)
Total	53 (6.1%)	249 (28.7%)	544 (62.7%)	21 (2.4%)	867 (100%)

**Table 3:** YFV infection rate for *Haemagogus janthinomys* by area.

Sampled Areas	Infected/Sampled	Infection Rate
Area 1	0/13	0%
Area 2	1/11	9.9%
Area 3	1/109	0.9%
Area 4	11/25	44%
Total	13/158	8.2%

## Supplementary Materials

The following are available online at https://www.mdpi.com/2075-4450/10/5/136/s1, Table S1: Standard curve values, Table S2: Viral quantification and Ct value of the YFV positive samples, Table S3: Viral quantification and Ct values obtained after viral isolation.
