# Supplementary material for: Detection of Yellow Fever Virus in Sylvatic Mosquitoes during Disease Outbreaks of 2017–2018 in Minas Gerais State, Brazil
Source: Insects. 2019 May 10;10(5):136. doi: 10.3390/insects10050136 (PMC6572267; doi:10.3390/insects10050136)
Supplement: Supplementary file 1 [file insects-10-00136-s001.pdf]

insects

MDPI

Article

Detection of yellow fever virus in sylvatic mosquitoes during disease outbreaks of 2017–2018 in Minas Gerais State, Brazil

Guilherme Garcia Pinheiro [1,3,*], Marcele Neves Rocha [2], Maria Angélica de Oliveira [1], Luciano Andrade Moreira [2] and José Dilermando Andrade Filho [3,*]

1 Coleção de Mosquitos Neotropicais, Instituto René Rachou, Avenida Augusto de Lima, 1715, Belo Horizonte, 30190-002, Brazil; angelica.oliveira@fiocruz.br
2 Mosquitos Vetores: Endossimbiontes e Interação Patógeno-Vetor, Instituto René Rachou, Avenida Augusto de Lima, 1715, Belo Horizonte, 30190-002, Brazil; marcele.rocha@fiocruz.br (M.R.), luciano.andrade@fiocruz.br (L.M.)
3 Grupo de Estudos em Leishmanioses, Instituto René Rachou, Avenida Augusto de Lima, 1715, Belo Horizonte, 30190-002, Brazil
* Correspondence: ggarciapinheiro@gmail.com (G.P.), jose.andrade@fiocruz.br (J.A.); Tel.: +55-31-3349-7856 (G.P.); Tel.: +55-31-3349-7874 (J.A.)
Received: 25 February 2019; Accepted: date; Published: date

Supplementary Materials

Table S1. Standard curve values

Standard curve	Ct value	Concentration	Ct Mean	Concentration Mean
YFV 10^7	12.02	1.16×10^7	11.94	1.23×10^7
YFV 10^7	11.85	1.31×10^7		
YFV 10^6	15.52	9.11×10^5	15.56	8.85×10^5
YFV 10^6	15.6	8.59×10^5		
YFV10^5	18.64	9.45×10^4	18.68	9.19×10^4
YFV 10^5	18.72	8.92×10^4		
YFV 10^4	21.7	1.03×10^4	21.74	1.00×10^4
YFV 10^4	21.77	9.74×10^3		
YFV10^3	25.04	9.07×10^3	25.3	7.64×10^2
YFV10^3	25.56	6.22×10^2		
YFV10^2	28.03	1.04×10^2	27.68	1.38×10^2
YFV10^2	27.33	1.72×10^2		

Table S2. Viral quantification and Ct value of the YFV positive samples.

Sample	YFV quantification (RT-qPCR)	Ct value
Hg. janthinomys area 2	7.90 x 10^7	18.40

Hg. janthinomys area 3	8.23×10^7	18.34
Hg. janthinomys area 4 (1)	3.63×10^8	16.16
Hg. janthinomys area 4 (2)	1.79×10^8	17.25
Hg. janthinomys area 4 (3)	1.44×10^7	20.90
Hg. janthinomys area 4 (4)	4.29×10^7	19.32
Hg. janthinomys area 4 (5)	7.89×10^7	18.40
Hg. janthinomys area 4 (6)	1.44×10^9	14.13
Hg. janthinomys area 4 (7)	1.84×10^8	17.16
Hg. janthinomys area 4 (8)	1.56×10^8	17.41
Hg. janthinomys area 4 (9)	7.50×10^7	18.48
Hg. janthinomys area 4 (10)	2.71×10^7	20.05
Hg. janthinomys area 4 (11)	3.42×10^7	19.64

Table S3. Viral quantification and Ct values obtained after viral isolation.

Sample	7 dpi		10 dpi	
	RT-qPCR	Ct value	RT-qPCR	Ct value
Hg. janthinomys area 2	1.46×10^9	15.52	4.42×10^9	14.01
Hg. janthinomys area 3	2.58×10^8	17.91	1.42×10^8	18.73
Hg. janthinomys area 4 (1)	9.51×10^8	16.14	1.11×10^{10}	12.73
Hg. janthinomys area 4 (2)	4.84×10^8	17.05	4.90×10^9	13.86
Hg. janthinomys area 4 (3)	5.68×10^6	23.17	2.41×10^9	14.83
Hg. janthinomys area 4 (4)	3.26×10^8	17.59	4.71×10^9	13.91
Hg. janthinomys area 4 (5)	2.95×10^9	14.55	1.31×10^{10}	12.50
Hg. janthinomys area 4 (6)	1.03×10^9	16.00	3.12×10^9	14.48
Hg. janthinomys area 4 (7)	3.66×10^9	14.25	6.18×10^9	13.53
Hg. janthinomys area 4 (8)	2.89×10^9	14.59	7.46×10^9	13.29
Hg. janthinomys area 4 (9)	2.17×10^9	14.97	4.73×10^9	13.90
Hg. janthinomys area 4 (10)	1.42×10^8	18.73	9.88×10^8	16.06
Hg. janthinomys area 4 (11)	6.80×10^6	22.92	1.30×10^7	22.03

© 2019 by the authors. Submitted for possible open access publication under the terms and conditions of the Creative Commons Attribution (CC BY) license (http://creativecommons.org/licenses/by/4.0/).
